# Relationship Between Short Term Variability (STV) and Onset of Cerebral Hemorrhage at Ischemia–Reperfusion Load in Fetal Growth Restricted (FGR) Mice

**DOI:** 10.3389/fphys.2018.00478

**Published:** 2018-05-18

**Authors:** Takahiro Minato, Takuya Ito, Yoshiyuki Kasahara, Sayaka Ooshio, Tomofumi Fushima, Akiyo Sekimoto, Nobuyuki Takahashi, Nobuo Yaegashi, Yoshitaka Kimura

**Affiliations:** ^1^Advanced Interdisciplinary Biomedical Engineering, Tohoku University Graduate School of Medicine, Sendai, Japan; ^2^Center for Development of Advanced Medical Technology, Jichi Medical University, Shimotsuke, Japan; ^3^Division of Clinical Pharmacology and Therapeutics, Tohoku University Graduate School of Pharmaceutical Sciences and Faculty of Pharmaceutical Sciences, Tohoku University, Sendai, Japan; ^4^Department of Gynecology and Obstetrics, Tohoku University Graduate School of Medicine, Sendai, Japan

**Keywords:** fetal growth restriction (FGR), cardiac autonomic control, cerebral hemorrhage, fetal electrocardiogram, short term variability (STV)

## Abstract

Fetal growth restriction (FGR) is a risk factor exacerbating a poor neurological prognosis at birth. A disease exacerbating a poor neurological prognosis is cerebral palsy. One of the cause of this disease is cerebral hemorrhage including intraventricular hemorrhage. It is believed to be caused by an inability to autoregulate cerebral blood flow as well as immaturity of cerebral vessels. Therefore, if we can evaluate the function of autonomic nerve, cerebral hemorrhage risk can be predicted beforehand and appropriate delivery management may be possible. Here dysfunction of autonomic nerve in mouse FGR fetuses was evaluated and the relationship with cerebral hemorrhage incidence when applying hypoxic load to resemble the brain condition at the time of delivery was examined. Furthermore, FGR incidence on cerebral nerve development and differentiation was examined at the gene expression level. FGR model fetuses were prepared by ligating uterine arteries to reduce placental blood flow. To compare autonomic nerve function in FGR mice with that in control mice, fetal short term variability (STV) was measured from electrocardiograms. In the FGR group, a significant decrease in the STV was observed and dysfunction of cardiac autonomic control was confirmed. Among genes related to nerve development and differentiation, *Ntrk* and *Neuregulin 1*, which are necessary for neural differentiation and plasticity, were expressed at reduced levels in FGR fetuses. Under normal conditions, *Neurogenin 1* and *Neurogenin 2* are expressed mid-embryogenesis and are related to neural differentiation, but they are not expressed during late embryonic development. The expression of these two genes increased in FGR fetuses, suggesting that neural differentiation is delayed with FGR. Uterine and ovarian arteries were clipped and periodically opened to give a hypoxic load mimicking the time of labor, and the bleeding rate significantly increased in the FGR group. This suggests that FGR deteriorates cardiac autonomic control, which becomes a risk factor for cerebral hemorrhage onset at birth. This study demonstrated that cerebral hemorrhage risk may be evaluated before parturition for FGR management by evaluating the STV. Further, this study suggests that choosing an appropriate delivery timing and delivery method contributes to neurological prognosis improvement.

## Introduction

Prediction and prevention of cerebral palsy are important issues for obstetricians and neonatologists. Approximately 15% of cerebral palsy cases are attributed to hypoxia at birth (Nelson and Ellenberg, 1986). However, the risk of developing cerebral palsy increases when hypoxic stress at delivery is added to fetal growth restriction (FGR), intrauterine infection, or immaturity due to preterm birth (MacLennan et al., 2015). Periodic uterine contraction at birth reduces placental blood flow and places a hypoxic load on the fetus, and the blood volume reduced by relaxation is recovered. In other words, a periodic hypoxic load is applied to the fetus during labor. Cardiotocogram (CTG) was developed in the 1960s to monitor the condition of the fetus at birth. Monitoring fetal heart rate changes and the frequency and intensity of uterine contractions to detect fetal hypoxia and acidosis at an early stage and conducting intervention could lead to the prevention of cerebral palsy. However, there are reports that the false positive rate was high, and cesarean sections and vaginal instrument deliveries were increased, and overall perinatal mortality was not affected (Vintzileos et al., 1995). The frequency of cesarean sections at the time of delivery has increased by around six times while the frequency of cerebral palsy has been almost unchanged since the 1960s (Colver et al., 2014; MacLennan et al., 2015). It has also been reported that neonatal convulsions were decreased, but there was no effect on the frequency of maternal mortality and cerebral palsy (Alfirevic et al., 2006). Since CTG does not contribute to the neurological prognosis, a new parameter for improving prognosis is needed.

FGR is one of the risk factors for cerebral palsy. FGR increases the risk of cerebral palsy at delivery by around 30 times (Blair and Nelson, 2015). Placental hypoplasia, which is one of its main causes, reduces placental blood flow, such that sufficient oxygen cannot be provided to the fetus, leading to chronic hypoxia. Cerebral hemorrhage, which is one of the main causes of cerebral palsy, is common around the ventricle and intraventricular space. In addition to the immaturity of blood vessels, cerebral hemorrhage is caused by the lack of cerebral blood flow (Ballabh, 2014). Cerebral blood flow is maintained at a constant level by controlling vascular resistance and cardiac output of autonomic nerves. However, autonomic nerve function in FGR is thought to be decreased, and it is expected to be a risk factor for the onset of cerebral hemorrhage at birth. Therefore, evaluating the function of autonomic nerves in FGR is important for estimating the risk of cerebral hemorrhage. At present, however, there is no means to decide the delivery time of the fetus and the delivery method by evaluating autonomic nerve function of the fetus at the clinical site. Autonomic nerve system maintains cerebral blood flow by controlling vascular resistance and cardiac output. The medulla oblongata is the center of these nerves. Chemoreceptor and baroreceptor send the information about atrial blood gas, pH and blood pressure to the medulla oblongata that controls cardiac output and heart rate via autonomic nerve system to maintain the cerebral blood flow. Even in a rest state, small adjustment in heart rate are made and that results in fluctuation in heart rate. Short term variability (STV) is used to measure the cardiac autonomic control. This is an average of the absolute values of the differences between adjacent RR intervals in the electrocardiogram (Van Ravenswaaij-Arts et al., 1993).

This study thus aimed evaluate the effect of FGR on the STV using fetal electrocardiography. The relationship between STV in FGR and the risk of developing cerebral hemorrhage at birth was evaluated by applying an ischemia–reperfusion load periodically, which mimics the condition at the time of delivery. As a secondary objective, the influence of FGR on the expression of genes associated with autonomic nervous system development and differentiation was considered.

## Materials and Methods

### Outline

First, an FGR model was created. Mice at mid-pregnancy (fetal age 15.5 days) underwent laparotomy under anesthesia, and the uterine artery was ligated to induce fetal development failure due to reduced placental blood flow. For the control group mice, only laparotomy was performed and the abdomen was closed. Laparotomy was performed again under anesthesia 48 h later, and fetal electrocardiography was performed for mice in both groups. STV used for evaluating autonomic nerves was calculated from the fetal electrocardiographic waveform. Subsequently, by applying a periodic ischemia–reperfusion load mimicking uterine contractions at the time of delivery and comparing the incidence and severity of cerebral hemorrhage in each group, the relationship between the cardiac autonomic control (STV) and the onset of cerebral hemorrhage was examined during FGR.

### Animal Experiment

This study was carried out in accordance with the recommendations of Tohoku University (animal experiment approval number 2017 saido-003).

#### Creating a FGR Model Mouse

**FIGURE 1:**
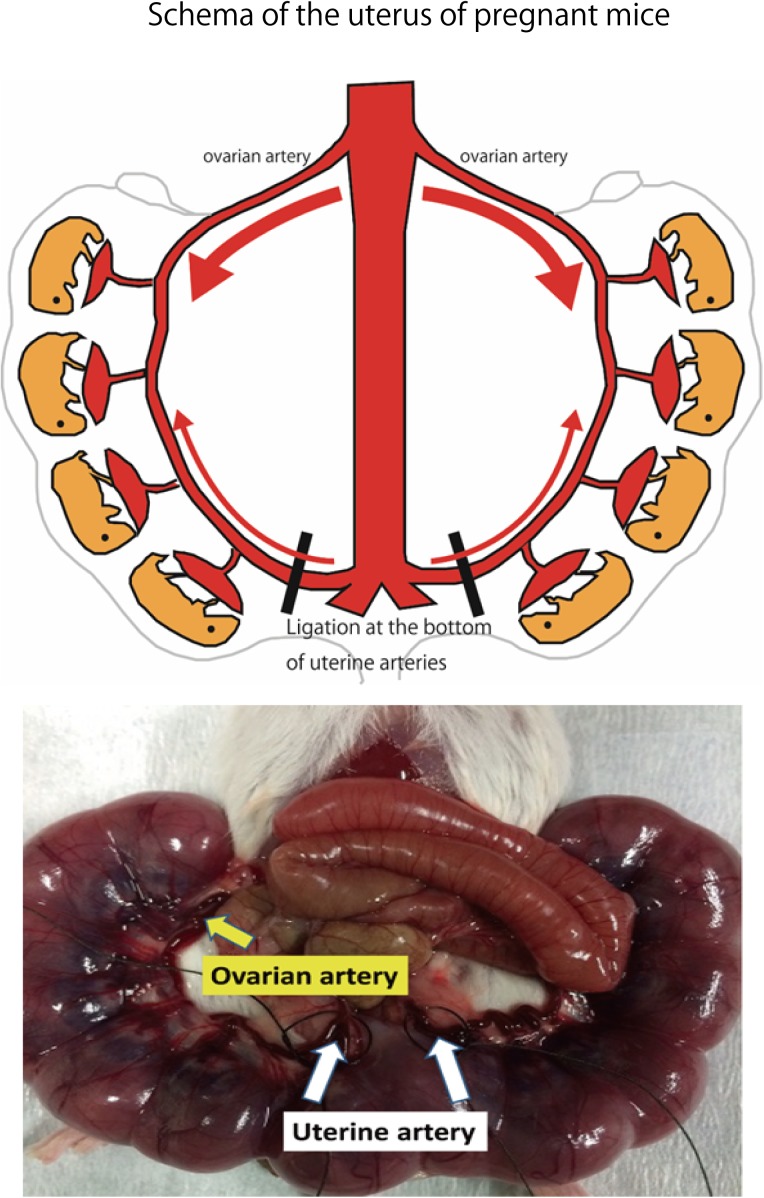
Schema of the uterus of pregnant mice. Bilateral uterine arteries are ligated to reduce the amount of oxygen supplied to the fetus by decreasing uteroplacental blood flow on day 15.5 of gestation. Image of uterine artery immediately before ligation. Area of the uterine artery that branches from the aorta to the uterine artery is ligated.

**FIGURE 2:**
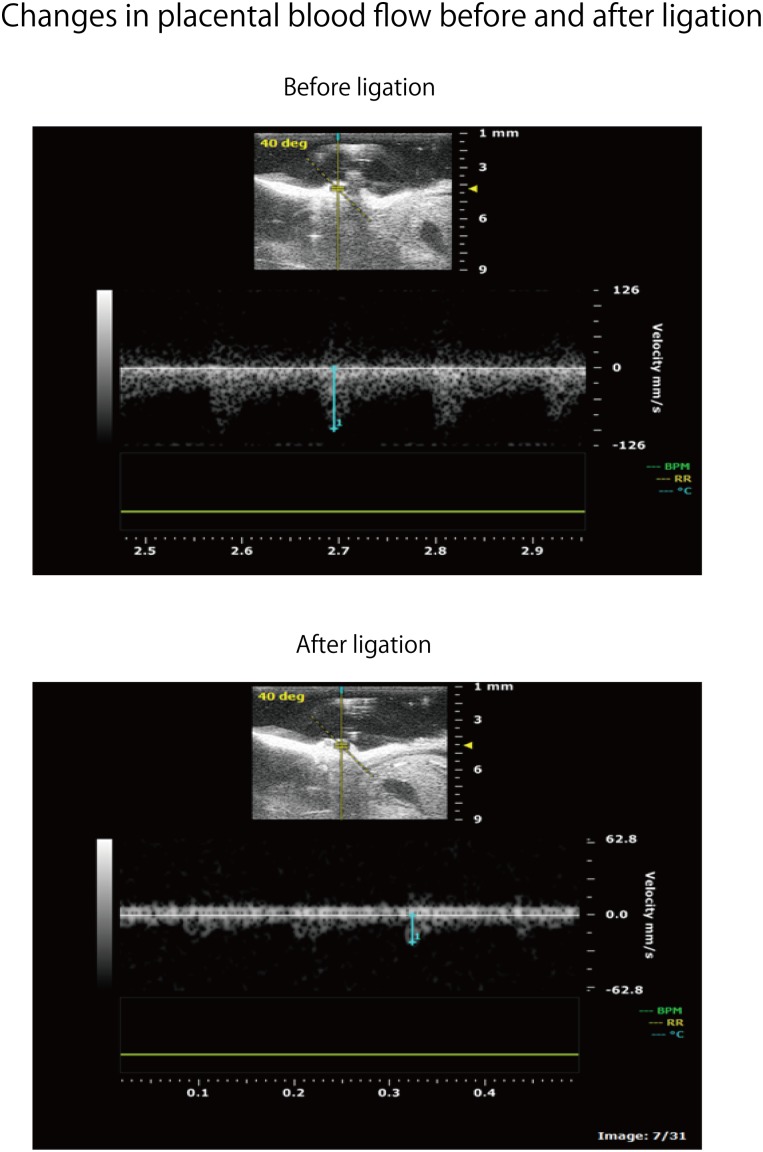
Flow rate change of uteroplacental blood flow before and after uterine artery ligation in the same spot. Strength of the ligature was controlled so as to be about half of the flow rate before ligature.

Adult ICR female mice at 8–12 weeks of age (non-pregnant weight: 27–35 g) were used. Female mice were mated overnight with age-matched male mice. If a vaginal plug was confirmed the next morning, that day was set as fetal age of 0.5 days. For confirming pregnancy, the weight change of mice was measured every day and an increase was confirmed. Mice had a dihedral uterus, and uteroplacental blood flow came from the ovarian arteries and uterine arteries (**Figure 1**). Fetal oxygen and nutrition are supplied via uteroplacental blood flow in the placental intervillous space. A median incision was made in the abdomen of mice under anesthesia on day 15.5 of gestation, and uteroplacental blood flow was decreased by ligation of the bilateral uterine artery (Fushima et al., 2016b), producing a chronic hypoxic load. For the induction of anesthesia, 0.2 ml of ketamine (Ketalar; Daiichi Sankyo Co., Ltd.) and 0.05 ml of xylazine (Seractar; Bayer Pharmaceutical Co., Ltd.) diluted to 1.0 ml with physiological saline were subcutaneously administered to pregnant mice at 0.05 ml/g. Anesthesia was maintained with isoflurane (Forane; Abbvie) inhaled at 0.5%. When the uterine arterial blood flow is completely blocked, the uteroplacental blood flow sharply decreases and all fetuses are miscarried at a high rate (Janot et al., 2014). Therefore, at the time of ligature, a 0.5-mm nylon thread was placed between the blood vessel and the ligature, and after ligating, the nylon thread was removed to give some clearance to avoid complete occlusion and a sudden decrease in blood flow. Furthermore, blood flow velocity of the uterine artery was monitored using an ultrasonic device (Vevo^®^ 2100; FUJIFILM VISUAL SONICS), and ligation was conducted by adjusting the flow rate to approximately half (**Figure 2**). Next, 0.0002 mg/g of isoxsuprine hydrochloride (Duvadilan; Daiichi Sankyo Co., Ltd.) was intraperitoneally administered for suppressing changes in blood flow due to uterine contraction during the experiment.

#### Measurement of Oxygen Concentration in Placental Tissue

A PO_2_ monitor (POG-301-1: UNIQUE MEDICAL Tokyo, Japan) was used for measuring oxygen partial pressure in placental tissue. On day 17.5 of pregnancy, mice in the FGR and control groups underwent laparotomy again. The position of the uteroplacental region was confirmed, and a 27-gauge indwelling needle was inserted approximately 3 mm from the uterine wall; it was confirmed by blood backflow that the tip was in the villous tissue of the placenta. The inner liner was removed, and the tip of the electrode was inserted to measure oxygen partial pressure in the placental tissue of mice in both groups.

#### Study on Fetal Hypoxia by Fluorescent Staining

It was confirmed that the fetus was under chronic hypoxia by fluorescent double staining of brain tissue with pimonidazole (Cosmo Bio; Hypoxyprobe^TM^) and hypoxia-inducible factor 1α antibody (Abcam; anti-HIF-1-α antibody). Pimonidazole is a chemical substance that binds to hypoxic cells, and HIF-1α is a transcription factor that accumulates in the nucleus under hypoxic conditions. Pimonidazole (0.006 ml/g, 50-fold dilution) was subcutaneously administered 40 min before re-laparotomy on day 17.5 of gestation. The number of mice used was one for each group (two mice in total). After re-laparotomy, the brain tissue of the fetus was excised after electrocardiography. The brain was quickly frozen with dry ice, and 8-μm coronal sections were prepared and fixed with 4% paraformaldehyde. Double staining using 4′,6-diamidiano-2-phenylindole and anti-HIF-1-α antibody was conducted. The primary antibody of HIF-1α was mouse anti-HIF-1α (1000-fold dilution), and the secondary antibody was goat anti-mouse IgG 555 (Alexa Fluor:^®^ diluted 500 times). The primary antibody of pimonidazole is FITC-Mab 1 (50-fold dilution).

#### Evaluation of the Influence of FGR on STV

Laparotomy was performed again under anesthesia on day 17.5 of pregnancy; an electrode was inserted into the uterus, and electrocardiography was performed (sampling rate: 1 kHz) to detect the QRS wave. The analysis target was waveform data of approximately 200 heartbeats without arrhythmia. LabChart Ver. 8 (ADInstruments Pty Ltd., Sydney, NSW, Australia) was used for the analysis. STV was used to measure the cardiac autonomic control. This is an average of the absolute values of the differences between adjacent RR intervals and is used as an index to evaluate the function of parasympathetic nerves in particular (Okai, 1984).

#### Study on the Effects of Uterine Artery Ligation on Fetal and Placental Weight

After electrocardiography, the fetus and placenta were excised from the uterus and weighed. Fetal body weight and placenta weight of mice in the FGR group were compared with those of mice in the control group, and the effect of chronic hypoxia on fetal growth was evaluated.

#### Consideration of Cerebral Hemorrhage Incidence and Bleeding Area for Ischemia–Reperfusion Load in the FGR Group

**FIGURE 3:**
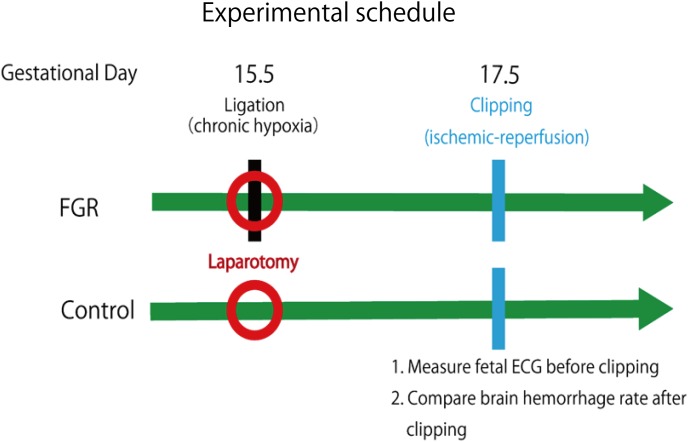
Experimental schedule of this research.

Ischemia–reperfusion loading due to uterine contraction during delivery was reproduced by temporarily blocking placental blood flow. In other words, it was reproduced by completely blocking blood flow of uterine and ovarian arteries using metal clips. Opening time and clipping time were set to 5 min each, in accordance with a previous experiment, and this was regarded as one cycle. Three cycles were conducted (Dong et al., 2014) (**Figure 3**). Then, brain tissue was removed and rapidly frozen with dry ice. Later, 8-μm coronal frozen brain slices were prepared, and the presence or absence of bleeding around the ventricle was examined (Dong et al., 2015). Bleeding area was measured in 30 different areas for each mouse, and the cerebral hemorrhage incidence and bleeding area were examined using Photoshop CC (Adobe Systems Incorporated, San Jose, CA, United States).

#### Comprehensive Analysis of Gene Expression

A comprehensive analysis of gene expression in the FGR group was performed, along with comparison with that in the control group. Mouse brain tissue at a fetal age of 17.5 days was excised and stored in RNAlater^TM^ Stabilization Solution (Thermo Fisher Scientific K.K., Yokohama, Japan), and mRNA was then extracted. Each group comprised six animals. Gene expression was compared by CAGE analysis (KK Dnaform, Yokohama, Japan, SRA accession number: SRP136767). We also used DAVID^1^ to analyze the gene ontology (GO).

### Statistical Analysis

Fetal body weight, placenta weight, placental tissue partial pressure, STV, RR interval, bleeding area and the number of bleeding spots were compared between the two groups. Before comparing these data, we used D’Agostino-Pearson Test to determine whether each data was normally distributed or not. We used an unpaired *t*-test when the data was normally distributed and when the data was not normally distributed, then we used Mann–Whitney test. Placental tissue partial pressure and RR interval were normally distributed. Fetal body weight, placenta weight, STV, bleeding area and the number of bleeding spots were not normally distributed. As for placenta weight and STV, these data were not normally distributed. A log transformation was performed. After applying the log transformation on the data, they exhibited normal distribution. After that, an unpaired *t*-test was performed for the data and the results showed significant difference. The distribution of those data were considered as log normal distribution. However, although the distribution of the data after performing log transformation was normal, the results showed no much difference from the Mann–Whitney *U*-test results. Hence, only Mann–Whitney *U*-test results are shown in the results. Data are expressed as mean ± standard error in unpaired *t*-test and are expressed as median value and *U* in Mann–Whitney test. To compare the incidence of cerebral hemorrhage between the two groups, the chi-square test was conducted. All statistical analyses were performed using GraphPad Prism ver. 6 (GraphPad Software Inc., La Jolla, CA, United States). In each test the significance level was set at *P* < 0.05.

## Results

### Influence of Ligation of the Bilateral Uterine Artery on the Fetus

**FIGURE 4:**
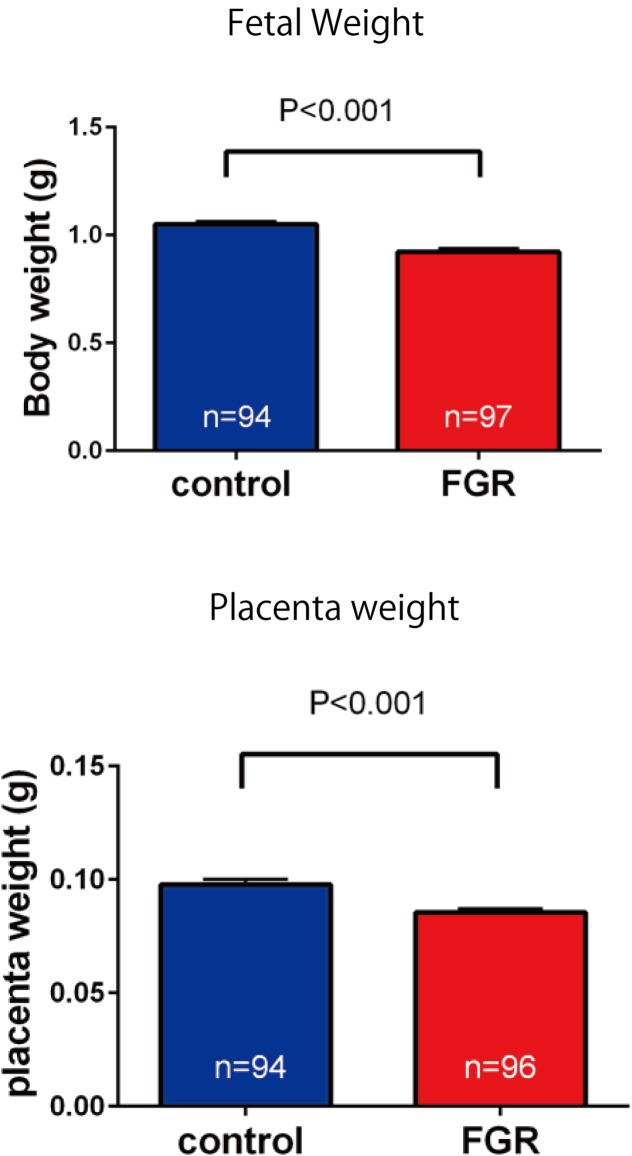
Comparison of fetal body weight and placenta weight. Both fetal weight and placenta weight were significantly decreased in the FGR group.

**FIGURE 5:**
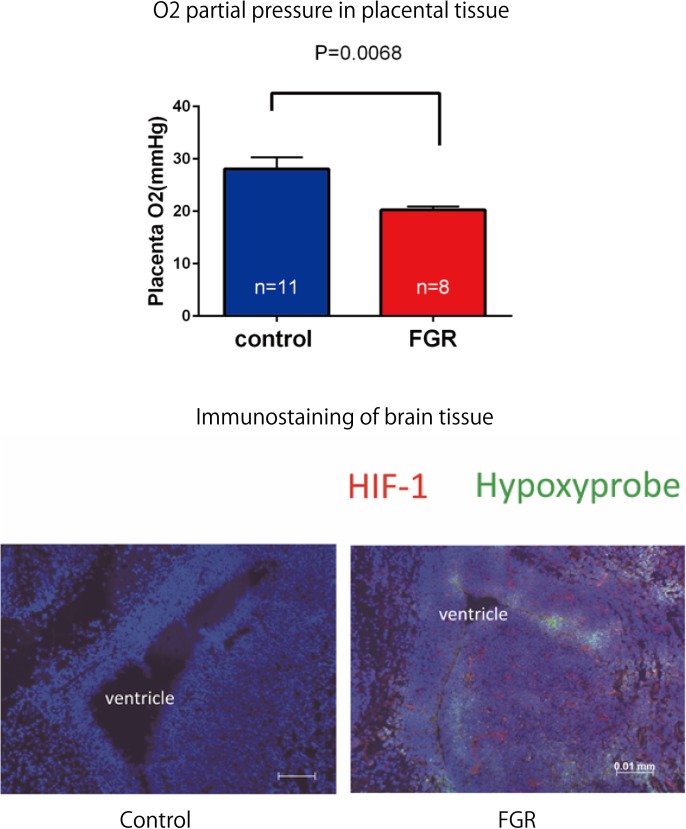
Oxygen partial pressure in placental tissues and fluorescent immunostaining of fetal brain tissue in both groups. Oxygen partial pressure decreased significantly in the FGR group. In fluorescent immunostaining, both HIF-1α and pimonidazole were negative in the control group, but in the FGR group both of these markers were positive.

Fetal body weight and placenta weight of mice the FGR group were compared with those of mice in the control group on day 17.5 of gestation. Fetal body weight significantly decreased in the FGR group (FGR group: 0.954 g, control group: 1.027 g, *U* = 2591, *P* < 0.001 Mann–Whitney test). Placenta weight also significantly decreased in the FGR group (FGR group: 0.083 g, control group: 0.093g, *U* = 3252, *P*-value < 0.001 Mann–Whitney test) (**Figure 4**). Oxygen partial pressure measurement in placental tissue and fluorescent immunostaining related to hypoxia in fetal brain tissue were performed. Regarding oxygen partial pressure in the placental tissue, the FGR group showed a significant decrease (FGR group: 20.2 ± 0.65 mmHg, control group: 28.1 ± 2.25 mmHg, *P* = 0.0068 unpaired *t*-test). For fluorescent immunostaining related to hypoxia, both HIF-1α and pimonidazole were positive in the FGR group, but both were negative in the control group (**Figure 5**).

### Influence of FGR on STV

**FIGURE 6:**
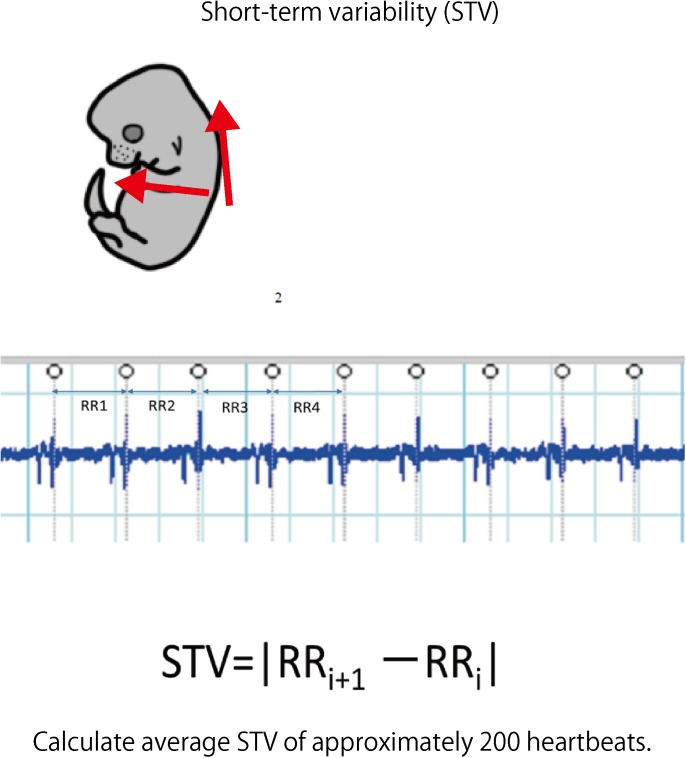
Fetal electrocardiography was performed using hyperbolic induction from the anterior chest and back. The STV of each fetus was calculated from about 200 heartbeats of the electrocardiogram waveform.

**FIGURE 7:**
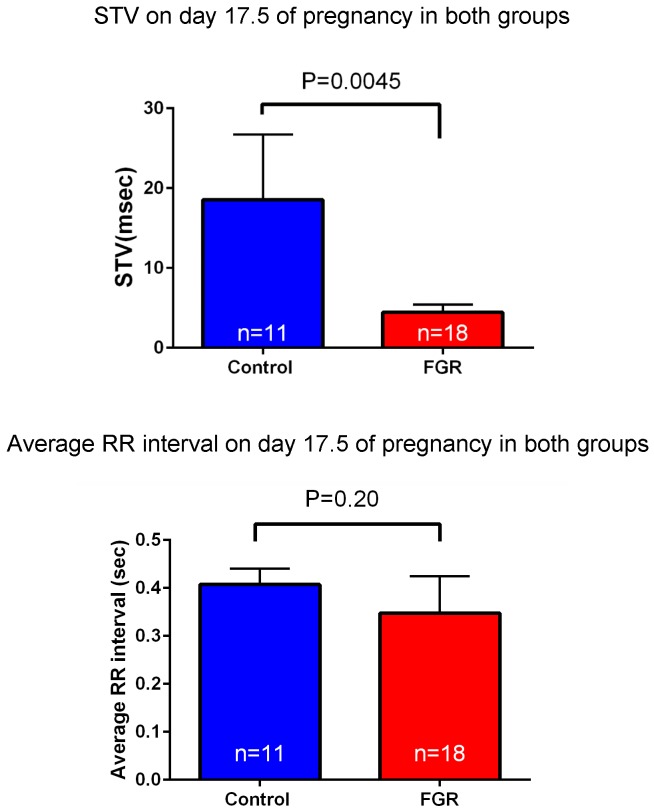
Heart rate and STV at 17.5 days pregnancy were compared between the two groups. There was no significant difference in heart rate between the two groups by uterine artery ligation. On the other hand, STV was significantly decreased in the FGR group.

Laparotomy was conducted again on day 17.5 of gestation, and fetal electrocardiography was also performed. We extracted the R wave and measured the STV, representing heart rate and autonomic nerve activity. Analysis was carried out for each fetus for about 200 heartbeats in the absence of clear tachycardia, bradycardia, and arrhythmia (**Figure 6**). The average of RR interval was 0.348 ± 0.018 sec in the FGR group and 0.407 ± 0.034 sec in the control group, with no significant difference between them (unpaired *t*-test). On the other hand, STV was 3.31 msec in the FGR group and 7.58 msec in the control group, which was significantly lower in the FGR group than in the control group (*U* = 37.5, *P*-value: 0.0045 Mann–Whitney test) (**Figure 7**).

### Examination of Cerebral Hemorrhage Incidence and Severity of Ischemia–Reperfusion Load in FGR Group

**FIGURE 8:**
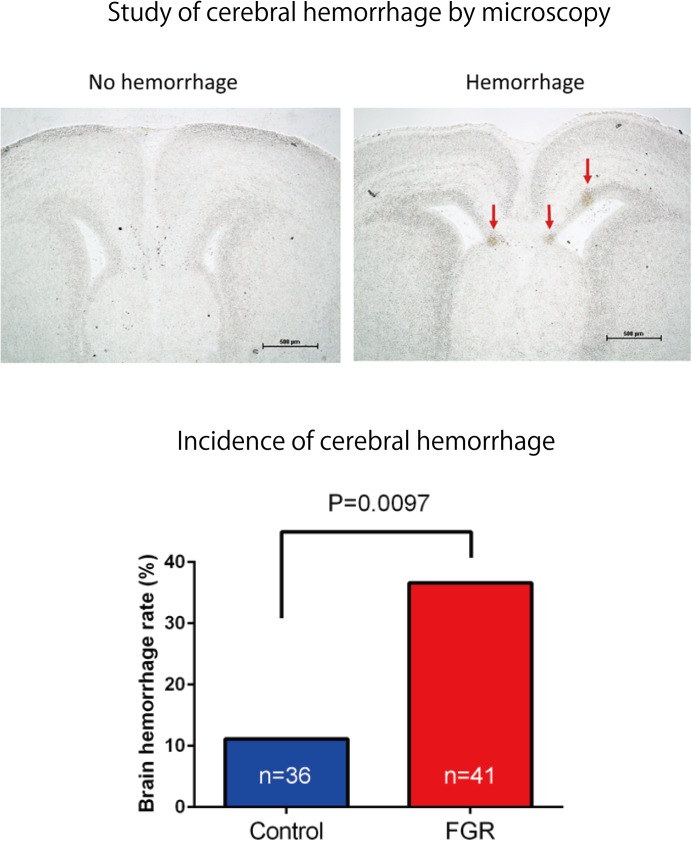
Frozen sections of brain tissue were prepared after ischemia–reperfusion, and the incidence of cerebral hemorrhage in each group was examined. There was a significant increase in the incidence of cerebral hemorrhage in the FGR group.

**FIGURE 9:**
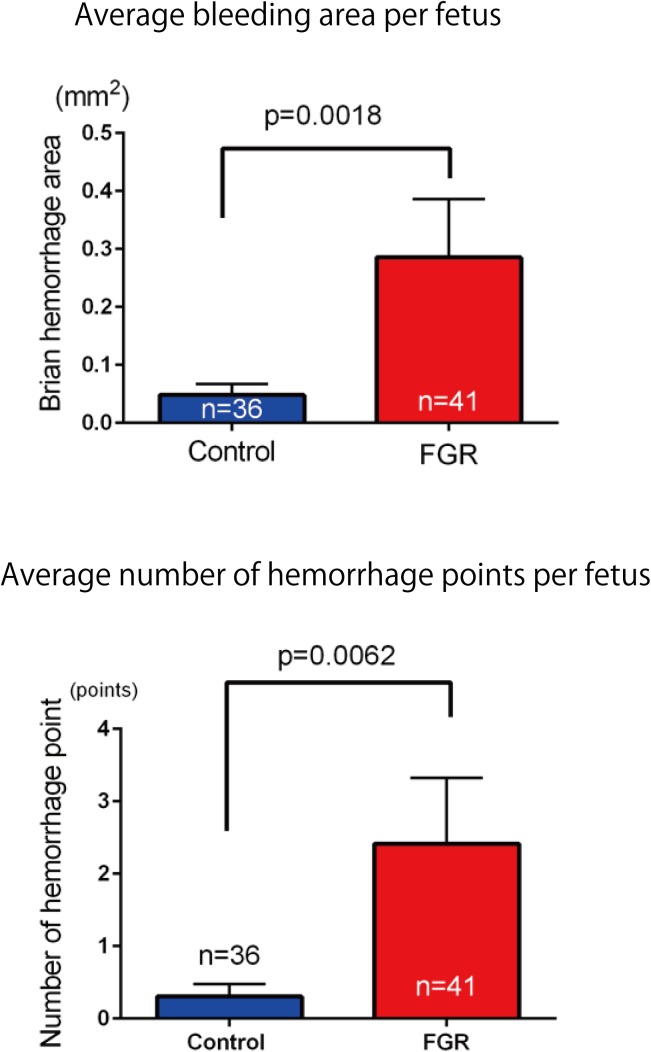
Investigation of average hemorrhage area and the number of hemorrhage points per fetus in both groups. In the FGR group, mean hemorrhage area and the number of hemorrhages also increased significantly compared with those in the control group.

The frequency and severity of cerebral hemorrhage upon periodic ischemia–reperfusion loading mimicking the conditions in delivery were compared between the two groups. In the FGR group, the incidence of cerebral hemorrhage was significantly increased compared with that in the control group (FGR group: 36.6%, control group: 11.1%, *P*-value: 0.0097) (**Figure 8**). The bleeding area and the number of bleeding spots per fetus were also examined to determine the severity of cerebral hemorrhage. In terms of the bleeding area, more extensive bleeding was observed in the FGR group than in the control group (FGR group: 0.60 mm^2^/mouse, control group: 0.00 mm^2^/mouse, *U* = 464, *P*-value: 0.0018 Mann–Whitney test). The number of bleeding spots was also significantly higher in the FGR group than in the control group (FGR group: 0.0 points/mouse, control group: 0.0 points/mouse, *U* = 542, *P*-value: 0.0062 Mann–Whitney test) (**Figure 9**). However the median value was the same between two groups, there were more hemorrhage points in FGR in average.

### Comprehensive Analysis of mRNA Expression Level

**FIGURE 10:**
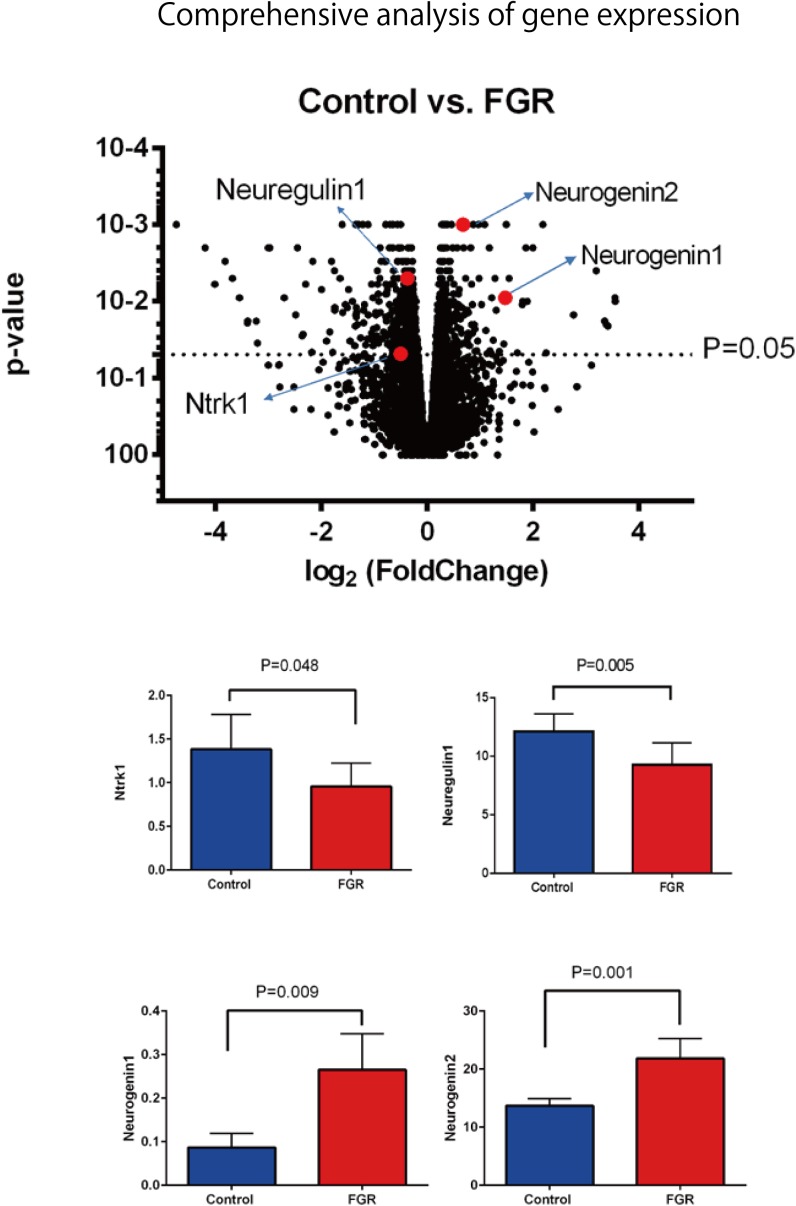
Comprehensive analysis of gene expression associated with FGR in brain tissue. Among the genes involved in nerve development, *Ntrk1* and *Neuregulin 1* showed significant decreases in expression, while *Neurogenin 1* and *Neurogenin 2* showed significant increases.

Our comprehensive analysis demonstrated that there were four genes that showed significant differences in expression level among the genes related to nervous system development and differentiation. Expression was significantly increased in *Neurogenin 1* (logFC: 1.482) and *Neurogenin 2* (logFC: 0.682), and significantly decreased in *Neuregulin 1* (logFC: -0.369) and *Ntrk 1* (logFC: -0.498) (**Figure 10**). The expression of Hist1h2ao was most increased compared with the control group. Hbb-1 and Alas2 that are responsible for the formation of hemoglobin are more expressed than in control group (**Table 1**). In the **Table 2**, five genes out of ten are the genes that are responsible for the structure of the histone protein. The number of GO terms that showed significant difference in expression is 44 (**Table 3** and Supplementary Data S1).

**Table 1:** The top 10 genes that show significant increases.

Gene name	logFC	logCPM	*P*-value
Hist1h2ao	2.856	5.885	*P* < 0.001
Gm3699	2.828	7.483	*P* < 0.001
Gm16869	2.781	2.189	*P* < 0.001
Rpl15-ps3	2.276	4.51	*P* < 0.001
Gm10801	1.495	8.039	0.001
Msl2	1.094	2.003	0.001
Hbb-b1	1.055	10.253	*P* < 0.001
Alas2	1.052	3.234	*P* < 0.001
Gm9833	1.044	5.08	*P* < 0.001
Gm10106	1.033	2.856	0.011

**Table 2:** The top 10 genes that show significant decreases.

Gene name	logFC	logCPM	*P*-value
Gm6363	-10.373	2.158	*P* < 0.001
Hist1h2ap	-8.74	8.718	*P* < 0.001
Hist2h3c2	-8.35	6.598	*P* < 0.001
Hist1h2br	-8.285	8.442	*P* < 0.001
Gm10243	-7.042	6.276	*P* < 0.001
Rpl17-ps3	-6.397	5.614	*P* < 0.001
Hist2h3c1	-4.743	6.214	*P* < 0.001
Dynlt1c	-3.985	1.843	*P* < 0.001
Rps26-ps1	-2.799	5.521	*P* < 0.001
Hist1h4k	-2.612	5.504	*P* < 0.001

**Table 3:** Gene ontology analysis.

Term	*P*-value
Nucleus	*P* < 0.001
Metal-binding	*P* < 0.001
Protein kinase, ATP binding site	*P* < 0.001
Extracellular matrix	*P* < 0.001
Hemoglobin complex	*P* < 0.001
Glycoprotein	*P* < 0.001
Protein tyrosine kinase activity	*P* < 0.001
Regulation of transcription from RNA polymerase II promoter	*P* < 0.001
repeat:ANK 3	*P* < 0.001
Nuclear chromosome	*P* < 0.001
TNF signaling pathway	*P* < 0.001
Insulin-like growth factor binding protein, N-terminal	0.001
Thrombospondin type 1 repeat	0.001
Histone methylation	0.002
Endoplasmic reticulum	0.003

*The TOP 15 categories that show significant change.*

## Discussion

**FIGURE 11:**
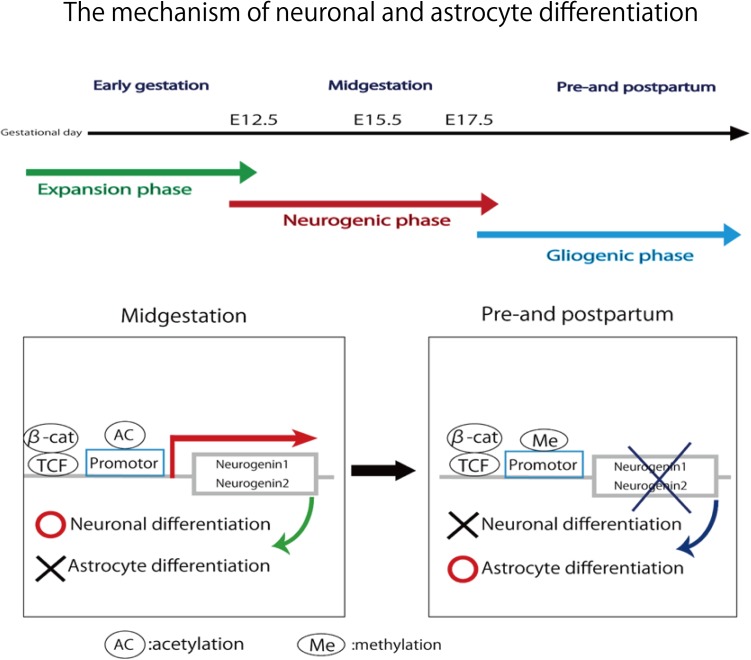
In midgestation, *Neurogenin1* and *Neurogenin2* promote neuronal differentiation and inhibit astrocyte differentiation. In pre- and post-partum, the expression of two genes are repressed due to the methylation of their promotor. Neuronal differentiation is inhibited and astrocyte differentiation is promoted.

In this study, pregnant mice were used to prepare an FGR model to which chronic hypoxic loading was applied. In this model, STV that is an indicator of cardiac autonomic control was decreased compared with the state in the control group. And the incidence of cerebral hemorrhage with ischemia–reperfusion loading, which occurs at the time of delivery, was significantly higher than in the control group. This suggests that dysfunction of cardiac autonomic control is a risk factor for cerebral hemorrhage. This also suggests that the risk of developing cerebral hemorrhage is predictable in advance by evaluating the STV by electrocardiography. In this experimental system, the uterine artery is ligated and the placental blood flow is decreased, which involves chronic hypoxic loading being applied to the fetus. The mother is known to develop pregnancy hypertension in this system (Li et al., 2012; Fushima et al., 2016a). Fushima et al. (2016b) who are collaborators in the present study, have demonstrated that the mother becomes hypertensive and that the placenta becomes hypoxic. Therefore, this model also involves pregnancy-induced hypertension as well as fetal FGR due to placental hypoplasia. Our results suggest that FGR is associated with a high risk of cerebral hemorrhage at birth, and dysfunction of cardiac autonomic control is considered to be one of the causes of this. To prove the occurrence of chronic hypoxia, we analyzed fetal blood gas, but since the mouse fetus was as small as about 1 cm, a suitable amount of blood for analysis could not be collected. However, since a decrease in uteroplacental blood flow was observed, the oxygen partial pressure in the placental tissue was lower than that in the control group, and hypoxic markers such as HIF1-α and pimonidazole were positive in the fluorescent immunostaining of fetal brain tissue, we concluded that the fetus itself had undergone chronic hypoxic loading. The effect of FGR on gene expression of brain tissue was also evaluated using comprehensive analysis. Hbb-1 and Alas2 that are responsible for the formation of hemoglobin were more expressed than in control group (**Table 1**). In GO analysis, the genes that have relationship with “hemoglobin complex” changed its regulation in FGR group. We assumed that increased hemoglobin formation was a compensation for the chronic hypoxic condition to supply the oxygen more efficiently to the tissues. Chronic hypoxic condition also affected gene expression. Five genes out of ten that were responsible for the structure of the histone protein (**Table 2**). Histone is a protein that is a major component of the nucleosome units. The term “Histone methylation,” “Nuclear chromosome” and “Nucleus” in GO analysis might indicate that the structure of chromosomes had changed and gene expression were different from that of control group. One of the changes in GO terms we focused on “Protein tyrosine kinase activity.” Tyrosine kinase is the enzyme that specifically phosphorylate tyrosine residues in protein. It involves signaling transmission related to cell differentiation, proliferation, etc. We found that, among genes related to the development and differentiation of cranial nerves, the expression of only *Ntrk* and *Neuregulin 1* significantly decreased. *Ntrk* is a gene encoding tropomyosin receptor kinase A (TrkA). Nerve growth factor (NGF) binds to this receptor to form a dimer. This dimer acts on sensory neurons and sympathetic postganglionic neurons in the peripheral nervous system and acts specifically on the forebrain basal cholinergic neurons in the central nervous system that project to the cerebral cortex and hippocampus to promote neuronal differentiation and neuronal survival/function maintenance (Fagan et al., 1997). *Neuregulin 1* is a gene that encodes neuregulin 1 that interacts with Neu/Her2/ErbB 2 receptor to increase its tyrosine phosphorylation. *Neuregulin 1* is widely expressed in the nervous system during its development, and plays diverse roles, including in synaptic plasticity regulation, glial differentiation, and myelination (Mei and Xiong, 2008). On the other hand, *Neurogenin 1* and *Neurogenin 2* showed significant increases in expression in the FGR group. In GO terms these genes belong to “E-box binding.” An E-box is a site that transcription factor binds and regulate gene expression in neurons, muscles and other tissues (Massari and Murre, 2000). The two genes encode the transcription factors Neurogenin 1 and Neurogenin 2, which are responsible for the differentiation of neural stem cells into neurons and the suppression of differentiation into astrocytes (Sun et al., 2001). In mice, neural precursor cells differentiate into neurons by the actions of these transcription factors in the middle of the embryonic period (around 10 to 15 days after gestation), but thereafter mainly differentiate into glial cells such as astrocytes (Hirabayashi and Gotoh, 2010; **Figure 11**). The expression of *Neurogenin* in the FGR group was higher than that in the control group in the late fetal period, suggesting that the differentiation of nerves may be delayed compared with that in the normal differentiation process. Chronic hypoxic condition could made these genes expression change and that change might decrease STV. Furthermore, considering that *Neurogenin* inhibits the differentiation into astrocytes, which are a major component of the blood–brain barrier (BBB), it may contribute to the vulnerability of cerebral blood vessels along with the delayed differentiation of nerves.

Although vulnerability of blood vessels is associated with a high risk of cerebral hemorrhage, cerebral blood flow and blood pressure are normally regulated at constant levels by autonomic nerves. Sympathetic and parasympathetic nerves are distributed in cerebral blood vessels, and sympathetic nerves constrict cerebral blood vessels and reduce cerebral blood flow via norepinephrine. Parasympathetic nerves exhibit cerebral vasodilatory activity and increase cerebral blood flow via acetylcholine. The antagonistic actions of the two types of nerve ensure automatic adjustment to maintain cerebral blood flow and blood pressure at constant levels. On the other hand, when the function of autonomic nerve adjustment is weakened, its autoregulatory capacity does not work adequately and cerebral blood flow cannot be maintained. Therefore, brain cell failure and cerebral vascular endothelial dysfunction due to ischemia and cerebral blood vessel failure due to sudden blood pressure fluctuation during reperfusion are likely to occur. Mechanisms of brain damage also include deterioration of lesions caused by microglia, in which cytotoxicity is activated by excitatory amino acids or free radicals, in addition to impairment of cranial nerve cells themselves due to cerebral blood flow reduction and oxygen depletion (Millar et al., 2017). This also involves the activity of autonomic nerves, especially parasympathetic nerves. Furukawa and colleagues administered galantamine, an acetylcholinesterase inhibitor, to activate parasympathetic nerves in rats. It inhibits the infiltration of microglia into lesions and suppresses inflammatory reactions, which leads to reduced severity of brain damage due to ischemic hypoxia (Furukawa et al., 2013, 2014). This also suggests the importance of evaluating the activity of autonomic nerves in assessing the brain damage risk. Measurement of STV using fetal electrocardiogram is a method for evaluating autonomic nerve activity. In this study, the fluctuation per heartbeat is shown in msec, which is an important parameter as an indicator of autonomic nervous system function, especially that of the parasympathetic nervous system. STV is influenced by hypoxia, gestational age, fetal activity and so on. In this experiment, we think that chronic hypoxia decreases STV, but we cannot deny the possibility that other factor may influence that parameter. To minimize the influence of other factor, we measure the STV when the fetus is at rest without fetal activity and respiratory movement. We use isoxsuprine hydrochloride to avoid uterine contraction that affect the STV. This parameter has also been adapted for clinical use in adults. For example, it is clinically applied as a means of predicting the prognosis of myocardial infarction (Kleiger et al., 1987). In addition to this research, attempts have been made on fetal adaptation. Okai performed a chronic experiment using pregnant Shiba goats and showed that STV decreased as fetal acidosis progressed under hypoxic loading (Okai, 1984). Murotsuki et al. (1997) also measured STV in experiments using sheep fetuses and showed that cardiac autonomic control mature as fetal age advances and that control is reduced in FGR fetuses. And that is secondary to placental insufficiency and FGR (Murotsuki et al., 1997). In addition, Ito et al. (2010) gave a protein-restricted diet to pregnant mice and measured the STV of their fetuses on day 17.5 of gestation. They found that the STV tended to decrease compared with that in the control group. Moreover, they demonstrated that these fetuses were at high risk of cerebral hemorrhage upon ischemia–reperfusion loading (Funamoto et al., 2017).

In this study, we measured the STV of mouse fetuses using fetal electrocardiography, and were able to evaluate the cardiac autonomic control in FGR fetuses. Power spectral analysis is a very important tool to understand the status of autonomic nervous system activity. On the other hand, STV has been one of the most important index in clinical practice for detection of fetal hypoxic state in fetus. The result of significant difference of STV in the two groups is thought to be very important in the clinical practice. Added of that, this is suitable for the clinical situation that we are required for making prompt diagnosis and treating of fetal hypoxia. Of course, power spectral analysis of fetal heart rate variability is very important. But the relation between hypoxic condition and autonomic nervous activity is not clear in fetal mice experiment yet. At the same time, we examined changes in RR interval (i.e., heart rate), but no significant change was observed between the two groups. We found that CTG cannot evaluate autonomic nerves. It was also shown that FGR is associated with a high risk of developing cerebral hemorrhage at birth, and dysfunction of cardiac autonomic control is one of the causes of this. Our results demonstrated that it is possible to evaluate the risk of cerebral hemorrhage before parturition by evaluating STV, which may contribute to the management of FGR, such as by optimizing delivery time and delivery method.

## Author Contributions

TM, TI, and YKi designed the study approach and experiment. TM, TI, TF, AS, and NT collected the research material. TM, TI and YKi were responsible for analysis and interpretation of the data. TM wrote the manuscript with contributions from TI, YKa, SO, YKi, and NY. All authors read and approved the final manuscript.

## Conflict of Interest Statement

The authors declare that the research was conducted in the absence of any commercial or financial relationships that could be construed as a potential conflict of interest.

## Footnotes

1 https://david.ncifcrf.gov/summary.jsp
