# Supplementary material for: Relationship Between Short Term Variability (STV) and Onset of Cerebral Hemorrhage at Ischemia–Reperfusion Load in Fetal Growth Restricted (FGR) Mice
Source: Front Physiol. 2018 May 18;9:478. doi: 10.3389/fphys.2018.00478 (PMC5968166; doi:10.3389/fphys.2018.00478)
Supplement: Supplementary file 1 [file Data_Sheet_1.docx]

Supplementary Data

Gene ontology analysis

| Term | Count | P-Value | Genes |
| --- | --- | --- | --- |
| Nucleus | 197 | 1.50E-07 | XRCC2, HIRA, RP9, VPRBP, CLK1, ZFP91, CLK2, CLK4, H2AFY, ZRSR1, SAP30L, FIZ1, HIST1H2BR, BARHL2, PTBP1, PIM1, NCAPD2, PRDM8, BAZ1A, JUN, MAPK3, VGLL2, EIF2AK2, SIVA1, NFKBID, TAL1, HEXIM2, PIR, FBXO3, ARNTL2, BCOR, LHX8, LHX9, KLF12, SMYD2, ZFP746, ZSCAN2, HNRNPA0, NOTCH2, PAXIP1, PCNA, SMURF2, TNK2, SPIN1, TAF1D, ZMAT3, MITF, GNL3L, YBX2, PBXIP1, ELOF1, NARF, FOXB2, THRSP, FGF3, BRD8, NFKBIZ, ZBTB20, HIST1H1B, VEZT, TRMT61A, TAF10, DACT1, FOXC2, MKNK2, HIST2H3C2, HIST2H3C1, STAU2, MLF1, CIR1, TCEA3, ZSCAN21, RB1CC1, ANKRD37, ETV5, MAPK11, ATRX, HIST1H2AP, TBATA, DUSP1, CSNK1E, FYTTD1, MEX3D, HIST1H2AL, NHLH1, HIST1H2AO, CHMP4B, CRABP2, CBX4, UBQLN4, CBX7, CTNNBL1, KLHL7, KDM1A, MUTYH, MIER2, SMARCD3, OIP5, UBXN2B, GATA3, RNF146, CIB1, GNL3, PABPN1, MAGEL2, NUDT1, MTA1, HDAC11, TRERF1, SIX6, JUNB, CDKL5, AHR, SUZ12, TNFAIP3, A230050P20RIK, HIST1H4K, SOX2, SOX4, MEIS1, SRC, KLHDC10, ZC3H8, HOXA3, SMARCB1, DYRK2, MFAP3L, ASF1B, DBX1, HIST1H4H, TMEM97, LPP, AKAP8L, NEUROG1, BRCA2, NR4A1, NEUROG2, MAFK, EHMT2, 2810417H13RIK, BRCA1, ZFP574, SALL2, PPIH, UACA, CDKN2AIP, PIP4K2A, HP1BP3, RAB3IP, AKT1, MEN1, FOS, ZFP647, PSMC3IP, POU4F2, POU4F1, PHC2, SSBP3, KHDRBS2, SOX13, ARID5A, IRF2BP2, WRAP53, PPM1H, UBC, CARM1, KPNA3, ZBTB8B, MGMT, BRSK1, DTNBP1, ZKSCAN16, N4BP1, TSPYL2, TSC22D4, KRT8, NFAT5, DCLK3, PLCD4, ZFP523, ZFP292, POU3F1, GAPDH, TERF2, CEBPA, CSRP2BP, MORN2, DACH1, ISL1, TAB2, TOX, MPG, RGS20, INVS, JAK2, ZFP536, MIF4GD |
| Metal-binding | 157 | 1.12E-07 | ADCY7, GBGT1, FAM20B, GNA12, NT5DC3, RP9, CAR5B, PRIM1, ZFP91, BAK1, HBQ1B, ZFP472, MUTYH, CDH20, ATP2B4, OIP5, GATA3, PHOSPHO1, HBB-B1, LONRF3, ZRSR1, HBB-B2, SAP30L, FIZ1, RNF146, CIB1, NQO2, NUDT1, FECH, NUDT12, PIM1, STIM2, MGP, MTA1, ZFP78, ZUFSP, TRERF1, MOXD1, MARK2, SUZ12, PRDM8, MIB1, PITPNM3, BAZ1A, ZFP598, TRIM32, RNF26, TNFAIP3, ADAMTS2, ADAMTS5, SIVA1, ZFP763, TIMP3, HBB-Y, RABGGTB, ZFP617, TIMP1, ZC3H8, ITGAV, PIR, LHX8, HBA-X, STK38L, LHX9, COL18A1, SCD1, S100A16, KLF12, LPP, AKAP8L, NR4A1, SMYD2, EHMT2, ZFP606, ZSCAN2, ZFP746, BRCA1, GAS6, ZFP574, DDX58, SALL2, TRIM56, MSL2, CDC42BPG, GM14325, PDZD8, FBXO30, CYP2R1, TNK2, PLA2G4E, GLRA1, ZMAT3, ATP10D, TDO2, CYP39A1, P4HA2, SMPDL3A, ELOF1, ZFP647, RMND5B, MT2, MT1, ATP8B2, CDK5RAP1, PHC2, ZBTB20, CAR13, CAPSL, CAR12, PRKCH, IRF2BP2, MMP15, MMP14, MAST2, CPXM1, FKBP14, CYBRD1, ADAM12, ALPL, DHH, ZBTB8B, FCNA, MGMT, BRSK2, MKNK2, CLCNKB, BRSK1, CDH3, CYB561D2, MTMR3, ZKSCAN16, SMOC2, TCEA3, ZSCAN21, ZFP523, PLCD4, ZFP292, BCKDHA, DCTD, DBH, ISL1, TAB2, MSRB2, HBA-A1, ATRX, GM14418, RASSF5, KCMF1, SUMF1, ZFP185, ACE2, MEX3D, JAK2, ZFP536, CDH10, ALOX8, ARAP1 |
| extracellular matrix | 26 | 1.94E-05 | FMOD, HIST1H4K, TIMP3, TIMP1, NOV, LGALS3BP, COL12A1, LAMB1, PCSK6, THBS2, GAPDH, CYR61, FN1, HIST1H4H, COL18A1, MATN2, MGP, MMP15, MMP14, COL14A1, BGN, SERPINF1, CMA1, MFAP4, ADAMTS2, ADAMTS5 |
| Protein kinase, ATP binding site | 33 | 1.88E-06 | MKNK2, BRSK2, CLK1, BRSK1, SRC, CSNK2A2, AKT1, CLK2, CLK4, DCLK3, DCLK2, DYRK2, STK38L, AATK, MET, PIM1, PRKCH, MAPK11, CDKL3, CDKL5, MARK2, NTRK3, MAP4K3, CDC42BPG, CSNK1E, EPHA8, NTRK1, MAPK3, GRK6, JAK2, TNK2, MERTK, EIF2AK2 |
| hemoglobin complex | 6 | 4.19E-05 | HBA-A1, HBQ1B, HBB-B1, HBB-B2, HBA-X, HBB-Y |
| repeat:ANK 3 | 17 | 3.16E-04 | NFKBIZ, OSTF1, NUDT12, NFKBID, PPP1R12C, TRPA1, EHMT2, KANK4, MIB1, NOTCH2, ZDHHC17, UACA, INVS, ANKS6, ANKRD37, TRPV4, BCOR |
| protein tyrosine kinase activity | 15 | 8.35E-05 | MET, CLK1, SRC, NTRK3, AKT1, CLK2, EPHA8, NTRK1, CLK4, JAK2, TNK2, DYRK2, MERTK, EIF2AK2, AATK |
| Glycoprotein | 158 | 6.19E-05 | SLC13A5, CADM4, ADCY7, GBGT1, FAM20B, TSPAN4, JAG2, CD52, HS2ST1, NOV, TTR, CDH20, HMCN1, COL12A1, UNC5C, 2510039O18RIK, SLC4A4, HIST1H2BR, MATN2, TRPA1, PLXNB3, STIM2, MOGS, CERCAM, PNPLA3, LPCAT3, SSPO, LPCAT4, MOXD1, SERPINF1, CD320, LAMC3, LPAR6, ROR1, MFAP4, EMP3, ADAMTS2, SLC40A1, GLP1R, HS3ST3B1, ADAMTS5, ENPP6, SCN1B, KCNA1, PTH1R, UNC93B1, IFI30, NKAIN1, FAM132A, ABCA1, TIMP1, LGALS3BP, FOLR1, BCHE, GPR27, ITGAV, NAGA, ENTPD4, MFAP3L, LAMB1, LRFN4, FN1, LRFN2, HCN1, COL18A1, MET, MCAM, TRIL, GAS6, ABCB4, NTRK3, NOTCH2, FAM198B, COL14A1, PPIA, EPHA8, NTRK1, ST8SIA5, SLC13A3, AREG, MERTK, ST7, GLRA1, SLC15A2, IGFBP6, SDC4, AKT1, SLC1A2, WISP1, P4HA2, SMPDL3A, CXCR4, SCRG1, GPC6, DMKN, PXMP4, ITIH5, SV2B, ITIH2, CHST15, LBP, TM9SF3, FGF3, HTR5A, SLC43A3, RAMP3, LAIR1, AVP, CAR12, KTN1, MMP15, CD164, SLIT3, BGN, CTSO, CPXM1, FKBP14, PLA2G7, CYBRD1, CTSC, ADAM12, CTSH, ALPL, GPR182, VIP, FMOD, GPRC5C, REN1, FCNA, ABHD2, CDH3, MFRP, VCAM1, CALCA, SMOC2, SLCO1A4, CD9, FNDC5, KRT8, PCSK9, SLCO3A1, PTX3, THBS2, NEFM, MPZ, PTPRZ1, PTPRA, FZD2, DBH, RGS20, SNED1, STAB1, LAYN, SUMF1, ACE2, CMA1, HTR2C, CDH10 |
| Regulationof transcription from RNA polymerase II promoter | 30 | 1.08E-04 | NFKBID, BBS7, MITF, SOX2, MEIS1, KDM1A, TAL1, FOS, ATP2B4, TCEA3, SMARCB1, GATA3, POU4F2, POU4F1, ARNTL2, POU3F1, ETV5, DBX1, CEBPA, NFKBIZ, BARHL2, RFX7, MAFK, ISL1, EHMT2, TRERF1, ANXA4, JUNB, AHR, NOTCH2 |
| Thrombospondintype 1 repeat | 9 | 0.001249197 | NOV, HMCN1, WISP1, UNC5C, THBS2, SSPO, ADAMTS2, ADAMTS5, CYR61 |
| Oxidoreductase | 32 | 0.00905745 | TM7SF2, CYB5R1, PRDX4, IFI30, KDM1A, TDO2, CYP39A1, P4HA2, FMO1, PIR, SRD5A3, GPX7, GAPDH, GFOD2, NQO2, SCD1, BCKDHA, RNLS, MTHFD2L, DHRS13, DECR1, DBH, MSRB2, MOXD1, DIO2, DIO3, SUMF1, PHGDH, CYBRD1, CYP2R1, CRYM, ALOX8 |
| transcriptional activator activity, RNA polymerase II core promoter proximal region sequence-specific binding | 19 | 0.006759581 | CEBPA, SSBP3, BARHL2, MITF, SOX2, NR4A1, SOX4, ISL1, MEIS1, JUNB, FOS, ZSCAN21, JUN, GATA3, NFAT5, FOXC2, POU4F2, POU4F1, ZFP292 |
| endoplasmic reticulum | 64 | 0.002678261 | TM7SF2, SLC27A1, GLRA1, SGPP2, CRABP2, PRDX4, UBQLN4, BAK1, FOS, ARHGAP5, SRPX, CYP39A1, P4HA2, UBXN2B, SLC35D3, SRD5A3, ATP8B2, GPX7, FGF3, DISC1, CIB1, CCDC88A, RINT1, STIM2, CST3, MGP, KTN1, MOGS, CERCAM, KRTCAP2, LPCAT3, LPCAT4, MOXD1, STOM, EBPL, NCK2, CD320, FKBP14, CTSC, APH1C, KCNA1, UNC93B1, BRSK2, DTNBP1, STAU2, SEC16B, VCAM1, FNDC5, BCHE, FMO1, PLCD4, PCSK9, AATK, H2-Q2, SCD1, TMEM97, FBXO2, S100A10, EPHX1, DBI, ITPR2, SDF2L1, SUMF1, CYP2R1 |
| domain: VWFA 1 | 4 | 0.019982428 | MATN2, VWA3A, COL14A1, COL12A1 |
| nuclear chromosome | 9 | 3.46E-04 | ATRX, HIST1H4K, BAZ1A, SMARCB1, HIST2H3C2, H2AFY, HIST2H3C1, HIST1H4H, NCAPD2 |
| Lipid metabolism | 22 | 0.019741341 | TM7SF2, SCD1, SLC27A1, ENPP6, CYB5R1, MCAT, ABHD2, DECR1, GPAT2, LPCAT3, PNPLA3, BRCA1, LPCAT4, MTMR3, CYP39A1, PLA2G7, PLCD4, PCSK9, ACAA1A, THRSP, PLA2G4E, ALOX8 |
| Insulin-like growth factor binding protein, N-terminal | 13 | 0.001043436 | MATN2, NOV, NOTCH2, HMCN1, WISP1, SNED1, STAB1, IGFBP6, JAG2, PCSK6, GAS6, SLIT3, CYR61 |
| Pathways in cancer | 23 | 0.01193272 | CEBPA, ADCY7, GNA12, MITF, MET, BRCA2, FZD2, COL4A5, AKT1, FOS, RASSF5, LPAR6, CXCR4, GNB1, LAMC3, ITGAV, JUN, NTRK1, MAPK3, PIK3CA, LAMB1, FGF3, FN1 |
| AGC-kinase,Cterminal | 6 | 0.027759293 | AKT1, CDC42BPG, MAST2, GRK6, PRKCH, STK38L |
| ferrous iron transmembrane transporter activity | 3 | 0.016620842 | SLC25A28, SLC25A37, SLC40A1 |
| Calcium　ion transmembrane transport | 8 | 0.030925789 | ATP2B4, ITGAV, CACNG7, TRPA1, TRPV4, CACNB3, GAS6, ITPR2 |
| Frizzled domain | 4 | 0.040992636 | COL18A1, ROR1, FZD2, MFRP |
| Ion transport | 29 | 0.028433356 | FXYD1, SCN1B, SLC13A5, GLRA1, SLC9A2, KCNA1, CLCNKB, CACNB3, KCNJ13, SLCO1A4, ATP2B4, TRPV4, SLC25A28, SLCO3A1, SLC4A4, HCN1, CACNG7, TRPA1, STIM2, NIPA1, KCTD2, ITPR2, CCDC109B, CLIC6, KCTD12B, SLC25A37, SLC41A1, SLC13A3, SLC40A1 |
| short sequence motif:Cell attachment site | 8 | 0.027496163 | COL18A1, COL14A1, COL12A1, PCSK9, MFAP4, THBS2, ADAMTS2, FN1 |
| Sodium/sulphate symporter | 3 | 0.009757452 | SLC13A5, SLC13A3, SLC13A4 |
| R-SMAD binding | 5 | 0.008959435 | MEN1, FOS, JUN, SMURF1, ARAP1 |
| Alpha carbonic anhydrase | 4 | 0.019049708 | CAR13, PTPRZ1, CAR12, CAR5B |
| E-box binding | 6 | 0.00613453 | TAL1, GATA3, NEUROG1, NEUROG2, ARNTL2, AHR |
| Tyrosine-protein kinase, catalytic domain | 8 | 0.01570111 | NTRK3, EPHA8, NTRK1, MET, JAK2, TNK2, MERTK, SRC |
| response to cAMP | 7 | 0.007590584 | FOS, REN1, DUSP1, JUN, AREG, CARM1, JUNB |
| ubiquitin-protein transferase activity | 19 | 0.037595517 | MAGEL2, UBE2G1, FBXO2, UBE2H, BRCA1, KLHL7, MIB1, ZFP91, TRIM56, KLHL8, RMND5B, TRIM32, FBXO30, SMURF2, FBXO3, SMURF1, TNFAIP3, BCOR, RNF146 |
| m_ace2Pathway:Angiotensin-converting enzyme 2 regulates heart function | 4 | 0.044795482 | REN1, ACE2, CMA1, COL4A5 |
| Cell junction | 30 | 0.037749314 | GLRA1, CCDC85C, KCNA1, BRSK1, DTNBP1, NOV, HMCN1, CXCR4, ITGAV, WNK4, TRPV4, SV2B, UNC5C, LRRC7, DISC1, LRFN2, DLGAP1, SVOP, LPP, VEZT, ARHGAP26, GJB2, DDX58, ZDHHC17, DACT1, MAST2, SVIL, ZFP185, CLDN2, TNK2 |
| m_edg1Pathway:Phospholipids as signalling intermediaries | 6 | 0.013084152 | AKT1, GNB1, ITGAV, MAPK3, PIK3CA, SRC |
| TNF signaling pathway | 12 | 9.01E-04 | AKT1, VCAM1, FOS, JUN, MAPK3, EDN1, PIK3CA, MAPK11, TNFAIP3, MMP14, TAB2, JUNB |
| Rotamase | 5 | 0.022076683 | PPIH, PPIA, GM12728, FKBP14, FKBP11 |
| active site:Glycyl thioester intermediate | 8 | 0.032832466 | ATG10, UBE2G1, UBE2L6, SMURF2, GM2058, SMURF1, GM10145, UBE2H |
| calcium ion transmembrane transport | 8 | 0.030925789 | ATP2B4, ITGAV, CACNG7, TRPA1, TRPV4, CACNB3, GAS6, ITPR2 |
| HAD-like domain | 7 | 0.044623498 | ATP2B4, PITPNM3, JAG2, NT5DC3, ATP8B2, PHOSPHO1, ATP10D |
| histone methylation | 6 | 0.001983537 | SUZ12, MEN1, PRDM8, SMYD2, CARM1, EHMT2 |
| short sequence motif:Cell attachment site | 8 | 0.027496163 | COL18A1, COL14A1, COL12A1, PCSK9, MFAP4, THBS2, ADAMTS2, FN1 |
| metal ion-binding site:Zinc; catalytic | 10 | 0.033530563 | DCTD, CAR13, CAR12, ACE2, CAR5B, MMP15, MMP14, ADAM12, ADAMTS2, ADAMTS5 |
| Zymogen | 13 | 0.032938867 | REN1, CTSO, PCSK9, CMA1, CTSC, MMP15, MMP14, ADAM12, CASP1, ADAMTS2, CASP2, CTSH, ADAMTS5 |
| Zinc-finger | 62 | 0.03526217 | ZMAT3, RP9, ZFP472, ZFP91, ELOF1, GATA3, ZFP647, RMND5B, ZRSR1, LONRF3, SAP30L, FIZ1, RNF146, PHC2, ZBTB20, MTA1, PRKCH, IRF2BP2, ZUFSP, ZFP78, TRERF1, PRDM8, SUZ12, MIB1, BAZ1A, ZFP598, TRIM32, RNF26, TNFAIP3, ZBTB8B, ZFP763, ZFP617, ZC3H8, ZKSCAN16, MTMR3, TCEA3, ZSCAN21, ZFP523, ZFP292, KLF12, AKAP8L, NR4A1, SMYD2, ZFP606, TAB2, ZSCAN2, ZFP746, BRCA1, ZFP574, GM14418, ATRX, MSL2, TRIM56, SALL2, CDC42BPG, RASSF5, GM14325, KCMF1, FBXO30, MEX3D, ZFP536, ARAP1 |
| membrane | 263 | 0.0104664 | CADM4, ADCY7, GBGT1, FAM20B, CD52, HS2ST1, PRIM1, BAK1, CDH20, ATP2B4, WNK4, 2510039O18RIK, LRRC7, TMEM14A, NCF4, RINT1, TRPA1, PTBP1, KRT10, LPCAT3, LPCAT4, MARK2, NCAPD2, EBPL, PITPNM3, CD320, LPAR6, ROR1, EIF2AK2, ADD3, MYH7B, HS3ST3B1, ENPP6, SCN1B, PTH1R, RAB40C, CACNB3, LGALS3BP, SCD1, FBXO2, TRIL, FXR2, MCAM, GPR153, NTRK3, NOTCH2, EPHA8, NTRK1, GRK6, COX6A2, CYP2R1, SMURF2, AREG, SMURF1, TNK2, PLA2G4E, TM7SF2, SLC27A1, GLRA1, SLC15A2, GNL3L, VPS37D, KCNJ13, POMGNT1, CYP39A1, TRPV4, CHST15, HTR5A, RAP2A, TMCO6, CACNG7, CAR12, VEZT, MMP15, CD164, RFTN1, MMP14, SLIT3, ZDHHC17, MAST2, CCDC109B, CLIC6, CYBRD1, ADAM12, FKBP11, ALPL, GPR182, DHH, RAB3D, REN1, CTXN3, HIST2H3C2, ABHD2, HIST2H3C1, STAU2, CYB561D2, SFT2D2, MTMR3, FNDC5, MPV17L2, FMO1, SLCO3A1, ACAA1A, AATK, DLGAP1, E130311K13RIK, COX7A1, PTPRZ1, FZD2, ITPR2, GJB2, STAB1, LAYN, SLC16A6, RPL21, SVIL, SLC16A9, PTP4A2, HTR2C, CDH10, CMTM5, TMEM176A, SLC13A5, CHMP4B, TSPAN4, GNA12, HBS1L, JAG2, GPAT2, CTNNBL1, SLC16A1, PACSIN2, SLC25A28, UNC5C, SLC4A4, DISC1, GNL3, CIB1, NUDT1, FECH, PLXNB3, STIM2, RPS6KC1, MOGS, PNPLA3, MOXD1, MIB1, VAMP8, RNF26, EMP3, SLC40A1, GLP1R, FXYD1, HIST1H4K, BBS7, TMEM216, BBS9, KCNA1, UNC93B1, NKAIN1, ABCA1, SRC, BCHE, GPR27, FOLR1, ITGAV, MFAP3L, STK38L, LRFN4, HIST1H4H, LRFN2, BEAN1, H2-Q2, HCN1, TMEM97, MET, EPHX1, NIPA1, ABCB4, DDX58, PLEKHA4, PDZD8, UACA, FAM198B, DIO2, PPIA, DIO3, ST8SIA5, SYT13, SLC13A3, PIP4K2A, MERTK, ST7, DENND5A, CYB5R1, SGPP2, SDC4, AKT1, NDUFS6, FOS, SLC1A2, ARHGAP5, CXCR4, SLC35D3, GPC6, PXMP4, SRD5A3, ATP8B2, SV2B, LBP, TM9SF3, CASP2, RAMP1, AHNAK, SLC43A3, RAMP3, LAIR1, SVOP, CCDC88A, AVL9, KTN1, MPP6, PRKCH, SLC9A3R1, KRTCAP2, SLC7A11, STOM, TMEM68, TMEM186, SLC35E1, CLDN2, SLC41A1, CTSC, GPRC5C, APH1C, FCNA, MGMT, CLCNKB, CDH3, DTNBP1, SEC16B, VCAM1, CD9, SLCO1A4, SHISA4, PLCD4, GAPDH, MTHFD2L, MPZ, H2-M3, DHRS13, SNX25, PTPRA, DBH, HBA-A1, RGS20, INVS, CCDC115, SDF2L1, ACE2, JAK2, ARAP1, ALOX8 |
